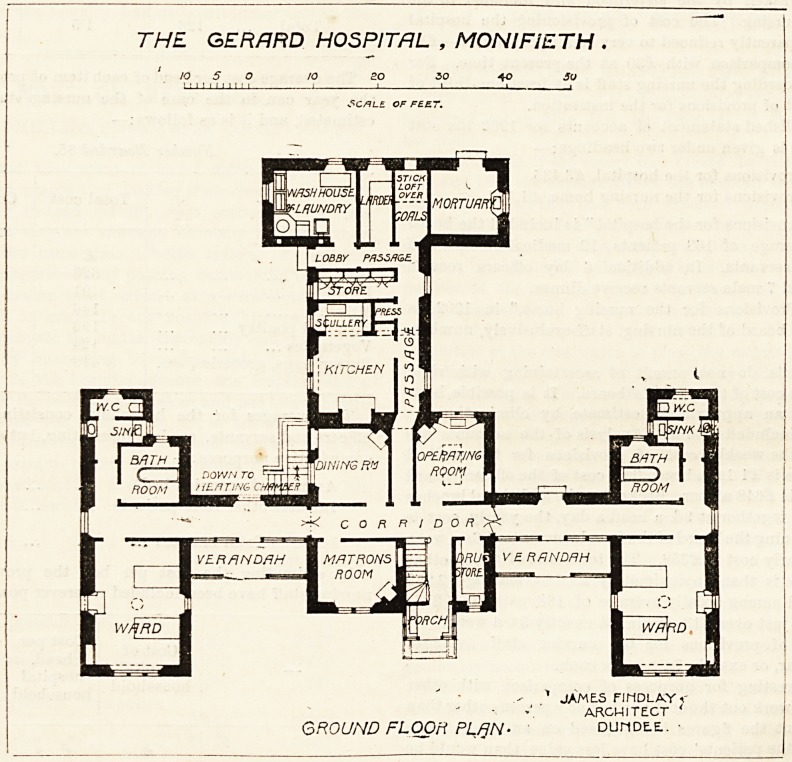# The Gerard Hospital, Monifieth, Forfarshire, N.B.

**Published:** 1904-06-11

**Authors:** 


### HOSPITAL ADMINISTRATION.

### CONSTRUCTION AND ECONOMICS.

### THE GERARD HOSPITAL, MONIFIETH, FORFARSHIRE, N.B.

The late Rev. J. Gerard Young, D.D., spent 45 years of his life as minister in this little town, and by his will left a sufficient sum not only to build, but to endow, the hospital, an instance of thoughtful generosity which it is pleasing to chronicle. The building was erected in due course, and was opened last year. It consists of an administrative centre, having to the front, entrance porch, matron's room, dispensary, and verandahs. Behind these is a corridor which gives access to the operation-room, the dining-room, and a passage leading to the kitchen and the laundry departments, and the mortuary. These departments are well arranged, though, as a rule, we should not place the larder next to the washhouse; but in so small a hospital this may, perhaps, pass without challenge. At each end of the main corridor, which presumably runs east and west, are the wards. Each is about 20 feet long and 12 feet wide, is intended for two beds, and therefore gives about 120 superficial feet per bed. There is no north point marked on the plan supplied to us; but if we guess rightly, the end of the ward faces south, which is the correct aspect, and in this end there is a large window in three sections. There is another window in the side of the ward, and opposite this one is the fireplace, with apparently a window over it. Whether this is sufficient for thorough perflation of air at all times may be doubted; but it must not be forgotten that the hospital is intended for a cold northern climate. Crossing the corridor, which is very well lighted, we come to the bath-room and closet, the latter being cut off by a cross-ventilated passage.

As an instance of planning, the Gerard Hospital is considerably in advance of the great majority of cottage hospitals we have seen, and with a little improvement in the cross-ventilation of the wards, which we have referred to, it would have been extremely good.

The warming is on the correct principle of open fire-places, assisted, when required, by hot water or hot air—we hope the former. The outer walls are whitewashed; the roofs are covered with red tiles and have broad eaves, giving the elevation a good appearance.

Miss Brebner, from the Dundee Royal Infirmary, was appointed matron, and it is reported that the institution is proving a great boon to the neighbourhood.

The architect was Mr. James Findlay, of Dundee. The cost is not stated in the particulars forwarded to us, which we regret, as with some modifications the plan might be a good model for similar hospitals, and the cost often plays an important part in these things.

## Figures and Tables

**Figure f1:**